# Multi-omics characterization of the microbial populations and chemical space composition of a water kefir fermentation

**DOI:** 10.3389/fmolb.2023.1223863

**Published:** 2023-10-02

**Authors:** Maria Clara Arrieta-Echeverri, Geysson Javier Fernandez, Adriana Duarte-Riveros, Javier Correa-Álvarez, Jorge Adalberto Bardales, Diego Fernando Villanueva-Mejía, Laura Sierra-Zapata

**Affiliations:** ^1^ Research Group CIBIOP, School of Applied Sciences and Engineering, Universidad EAFIT, Medellín, Antioquia, Colombia; ^2^ Infectious Diseases Biology and Control Group (BCEI), Universidad de Antioquia UdeA, Medellín, Colombia; ^3^ Iluma Innovation Labs, Iluma Alliance, Durham, NC, United States

**Keywords:** microbial communities, fermentation dynamics, probiotics, digestive health, multiomics approach, fermented food analysis

## Abstract

In recent years, the popularity of fermented foods has strongly increased based on their proven health benefits and the adoption of new trends among consumers. One of these health-promoting products is water kefir, which is a fermented sugary beverage based on kefir grains (symbiotic colonies of yeast, lactic acid and acetic acid bacteria). According to previous knowledge and the uniqueness of each water kefir fermentation, the following project aimed to explore the microbial and chemical composition of a water kefir fermentation and its microbial consortium, through the integration of culture-dependent methods, compositional metagenomics, and untargeted metabolomics. These methods were applied in two types of samples: fermentation grains (inoculum) and fermentation samples collected at different time points. A strains culture collection of ∼90 strains was established by means of culture-dependent methods, mainly consisting of individuals of *Pichia membranifaciens*, *Acetobacter orientalis*, *Lentilactobacillus hilgardii*, *Lacticaseibacillus paracasei*, *Acetobacter pomorum*, *Lentilactobacillus buchneri, Pichia kudriavzevii*, *Acetobacter pasteurianus*, *Schleiferilactobacillus harbinensis*, and *Kazachstania exigua*, which can be further studied for their use in synthetic consortia formulation. In addition, metabarcoding of each fermentation time was done by 16S and ITS sequencing for bacteria and yeast, respectively. The results show strong population shifts of the microbial community during the fermentation time course, with an enrichment of microbial groups after 72 h of fermentation. Metataxonomics results revealed *Lactobacillus* and *Acetobacter* as the dominant genera for lactic acid and acetic acid bacteria, whereas, for yeast, *P. membranifaciens* was the dominant species. In addition, correlation and systematic analyses of microbial growth patterns and metabolite richness allowed the recognition of metabolic enrichment points between 72 and 96 h and correlation between microbial groups and metabolite abundance (e.g., Bile acid conjugates and *Acetobacter tropicalis*). Metabolomic analysis also evidenced the production of bioactive compounds in this fermented matrix, which have been associated with biological activities, including antimicrobial and antioxidant. Interestingly, the chemical family of Isoschaftosides (C-glycosyl flavonoids) was also found, representing an important finding since this compound, with hepatoprotective and anti-inflammatory activity, had not been previously reported in this matrix. We conclude that the integration of microbial biodiversity, cultured species, and chemical data enables the identification of relevant microbial population patterns and the detection of specific points of enrichment during the fermentation process of a food matrix, which enables the future design of synthetic microbial consortia, which can be used as targeted probiotics for digestive and metabolic health.

## 1 Introduction

Microbial consortia are present in a wide range of environments including soils, biofilms, and food products, such as beer, kombucha, and dairy products (Padmaperuma et al., 2019). These associations play an important role in soil management and nutrient mobilization, and they have also been studied due to their potential in the industry and economic importance, as they are involved in the development of fermented foods, which confer nutritional properties to their consumers (Madigan et al., 2015; McCaughey et al., 2022). Fermented products have long been considered basic foods in many countries because the fermentation process is an old technique to produce, conserve, or transform the organoleptic properties of foods and beverages (Fiorda et al., 2017; Villarreal-Morales et al., 2018; Bengoa et al., 2019). Most of these matrices are spontaneous or can be fermented using a starter culture (culture-dependent ferments), so there are several variables in the process of their fermentation, including the source of microorganisms, their nutritional ingredients, and environmental conditions, resulting in thousands of different variations of these products (Villarreal-Morales et al., 2018; Dimidi et al., 2019; Sharma and Yaiphathoi, 2020; Safak et al., 2023).

In recent years, the popularity of these foods, including kombucha and kefir, has increased based on the potential health benefits that have been ascribed to them based on the metabiotics, which can be grouped into prebiotics, probiotics, and postbiotics produced by these products (Marco et al., 2020; Pihurov et al., 2023). These biological activities can be related to the production of biochemical reactions triggered by multiple microorganisms that result in the release of vitamins, amino acids, exopolysaccharides (EPS), and organic acids, among other bioactive compounds (Diez-Ozaeta and Astiazaran, 2022). Water kefir has been studied, mainly for its impact on the immune system and gastrointestinal health, being beneficial for preventing non-communicable diseases such as lactose malabsorption, diabetes, obesity, inflammation, and cardiovascular conditions through the modulation of gut microbiota (Fiorda et al., 2017; Dimidi et al., 2019; Calatayud et al., 2021; Araújo et al., 2023). The composition of water kefir is known to be a stable microbial community of lactic acid bacteria (LAB), acetic acid bacteria (AAB), and yeasts, as shown by both culture-dependent and culture-independent-based studies (Laureys and De Vuyst, 2014; Zanirati et al., 2015; Farag et al., 2020).

For many years, research projects on water kefir have relied on conventional culture-dependent methods consisting of the isolation and culturing of microbes prior to their identification according to either morphological, biochemical, or genetic characteristics (Jianzhong et al., 2009). These culture-dependent approaches aim to test different culture conditions (growth temperature, pH, carbon, and nitrogen source) that could help optimize culturing methods for target microbes in fermented food matrices such as water kefir (Wuyts et al., 2020). Furthermore, molecular culture-independent methodologies, specifically metabarcoding and shotgun sequencing, have proven to be a powerful tool to provide a more complete microbial diversity spectrum in food samples, especially for those microbial groups that are difficult to isolate by culture-dependent methods (Verce et al., 2019). Recent studies that have characterized the microbial populations of water kefir have found that the most common microorganisms are lactic acid bacteria of the *Lactobacillus*, *Leuconostoc,* and *Lactococcus* genus, acetic acid bacteria of the genus *Acetobacter* and *Gluconobacter,* and yeast such as *Saccharomyces cerevisiae* and *Zygotorulaspora florentina* (Gulitz et al., 2011; Fiorda et al., 2017; Chen et al., 2021; Spizzirri et al., 2023). Nevertheless, given the variability of substrates and water kefir grain origins, a diverse composition of species and strains can be found in each beverage (Moretti et al., 2022). For instance, the same report by Moretti et al. (2022) exemplifies the uniqueness of a WK beverage prepared in Antioquia, Colombia, which is called “*Arroz de indio*” or “*Indiecitos*”, based on the fermentation of “*Aguapanela*” or dry sugar cane solution, sharing some similarities with but not identical to the WK system of this study.

Furthermore, the health benefits of water kefir are not only related to the presence of certain microorganisms but also to the compounds produced during fermentation. Several studies have focused on determining the concentration of specific compounds including simple and short chain sugars, exopolysaccharides (EPS), organic acids, amino acids, and volatile compounds (Chen et al., 2021; Patel et al., 2022). Metabolite screening was carried out through different analytical platforms to perform targeted and untargeted studies such as gas chromatography/mass spectrometry (GC-MS), chromatography/mass spectrometry (GC/MS), liquid chromatography/mass spectrometry (LC/MS), and capillary electrophoresis/mass spectrometry (CE/MS). However, LC/MS has proven to deliver accurate qualitative and quantitative capability and provides the convenience of simultaneous multi-component analysis (Azi et al., 2021). For the water kefir biological system, most existing studies have been performed using targeted metabolomic techniques, the two most commonly used techniques being high-pressure liquid chromatogram coupled with triple quadrupole mass spectrometry (HPLC-MSMS) and headspace/solid phase microextraction coupled with gas chromatogram and mass spectrometry (HS/SPME-GC–MS) (Hu et al., 2014; Patel et al., 2022). These methods have resulted in the identification of bioactive compounds such as organic acids, amino acids, flavonoids, and phenols, which have been reported as health-promoting agents (Azi et al., 2021).

Based on the statements above, evidence suggests that understanding complex biological systems, such as water kefir, requires the application of multiple methods that provide different perspectives (Weckx et al., 2019). Accordingly, the following project aimed to explore and characterize the microbial chemical space of a water kefir fermentation by integrating three different -omics approaches. Also of interest when developing the study was identifying different points of microbial enrichment during fermentation, which can serve as the knowledge basis for the future rational design of synthetic consortia, with a potential application as a health-beneficial food supplement. This can be reached using the knowledge provided by this study on the WK matrix and the strains collection isolated during its development. We consider that this approach not only deciphers the microbial chemical diversity of a locally produced (in Colombia) and modified fermented food but also enables its further use as a therapeutic ingredient, such as in probiotics and postbiotics.

## 2 Materials and methods

### 2.1 Water kefir fermentation and sampling

**FIGURE 1:**
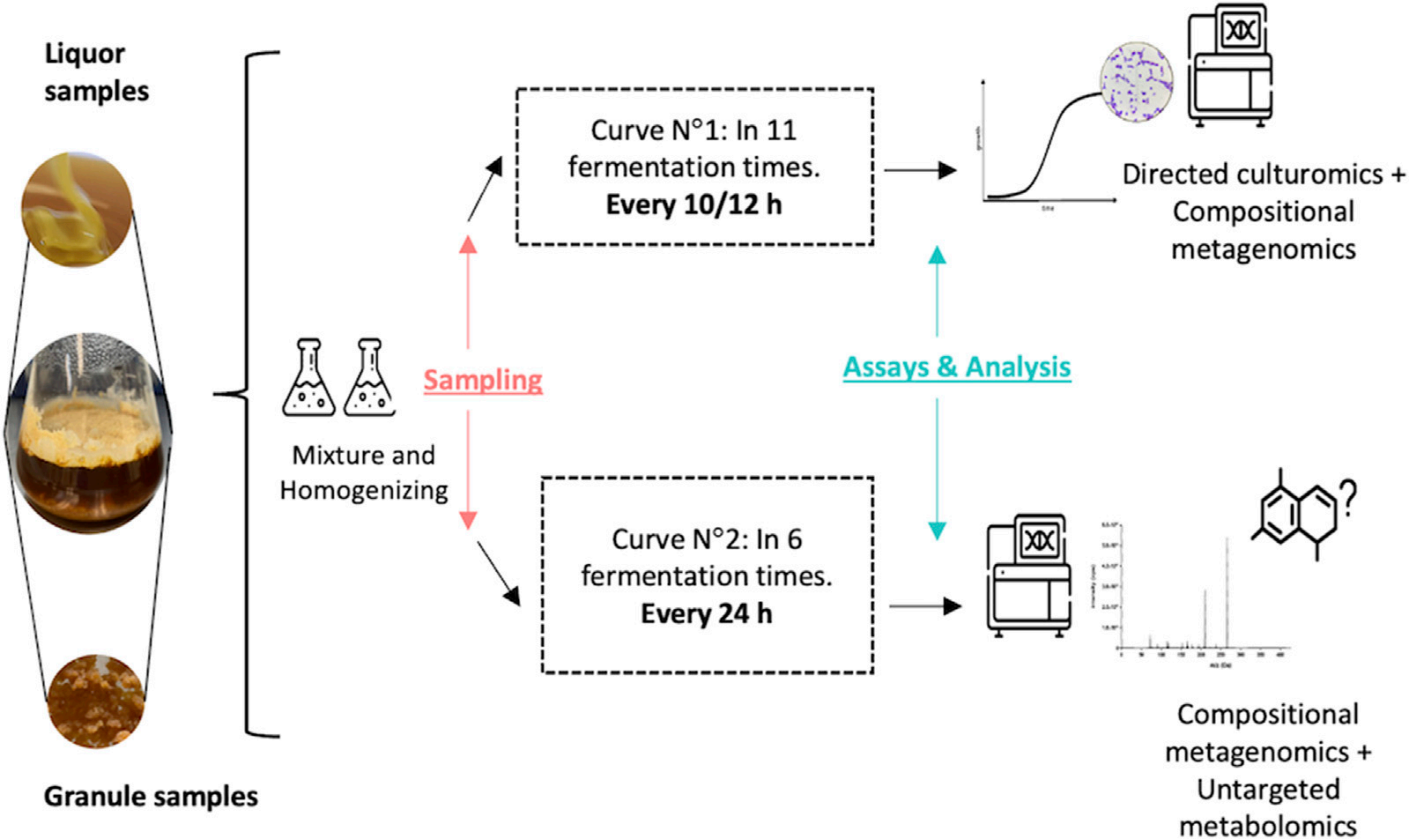
Experimental setup for microbial consortium sampling.

The water kefir fermentation was obtained from a private company in Colombia (Rionegro, Antioquia). As the origin of the sample remains unknown, the applied methods did not require the management of permits for the collection of biological material or access to genetic resources under the authorization of MinAmbiente (Colombia). Figure 1 shows the schematic diagram of the experimental setup. Two independent fermentation curves were performed using 12 g of kefir grains (which can be obtained for reproducibility assays upon request to the authors) in 100 mL of diluted molasses (14° Bx) on each sterile glass flask, then fermentation was set at room temperature (18°C) until the specific sampling time. Briefly, for the first curve, fermentation samples (grains and liquor) were collected every 10–12 h by duplicates, for 5 days, to perform microbial composition analysis and culturable-dependent population analysis. In total, 22 fermentation liquor samples (corresponding to data from 11 times by duplicates) and 2 inoculum (grains) samples were collected. The second growth curve was sampled every 24 h for 5 days (six different time points, in duplicates—14 samples), for the identification of non-culturable microorganisms and performing non-targeted metabolomic analysis (chemical space screening).

### 2.2 Culture-dependent methods

Three semi-selective and one non-selective culture media were chosen: MRS media, for lactic acid bacteria (62 g agar per L of distilled water liter (PanReac AppliChem, Darmstadt, Germany)); YM agar media for yeast (5 g of Peptone, 3 g Yeast extract, 3 g Malt extract, 10 g Dextrose, and 20 g Agar per L of distilled water, all substances from PanReac AppliChem, Darmstadt, Germany); WL, for acetic acid bacteria, (80,25 g per L of distilled water, Sigma-Aldrich, Saint Louis, United States); and MM, standardized culture media for total molasses degrading microbial biomass, which consist of diluted molasses to 14° Bx plus 15 g/L of bacteriological agar. Culture media were inoculated with 50 μL of serial dilutions of kefir grains homogenized with sterile 0.9% saline solution and liquor at different fermentation times (10^–2^, 10^–3^, 10^–4^) then incubated at room temperature (+/−25°C) for 72 h (3 days). Each dilution was cultured in duplicates. Afterward, colonies were counted in each dilution plate, differentiating by morphotypes, and registering CFU/mL for each culture media and fermentation time. Simultaneously, the isolation of representative morphotypes found in each culture media and fermentation time was registered. The purified morphotypes were Gram stained as a preliminary classification and then cryopreserved by duplicates at −80°C for downstream DNA extraction to constitute the first version of the strains collection derived from this microbial consortium.

### 2.3 Strains collection derived from representative morphotypes

Total DNA extraction from the resulting isolates was performed using the DNeasy Ultraclean Microbial Kit (QIAGEN, Hilden, Germany) for molecular identification through Sanger sequencing performed in the Macrogen Inc. sequencing facility (Seoul, South Korea). Primers used to perform the analysis were 27F (5′ AGAGTTTGATCMTGGCTCAG 3′) and 1492R (5′ TACGGYTACCTTGTTACGACTT 3′) for bacterial 16S rRNA hypervariable region (M. Y. Chen et al., 2022), and for yeast, the internal transcribed spacer ITS1 (5′ TCC​GTA​GGT​GAA​CCT​GCG​G 3′) and ITS4 (5′ TCC​TCC​GCT​TAT​TGA​TAT​GC 3′) (Taheur et al., 2017; Tan et al., 2022). Then, obtained data was uploaded to Geneious Prime ^®^ 2021.2.1 software (https://www.geneious.com) to perform sequence trimming, alignment, and finding of consensus for subsequent BLAST classification (using 100% accuracy parameter). Finally, the collection of purified strains was stored at the Laboratory Center of Universidad EAFIT in Medellín, Colombia.

### 2.4 Total DNA extraction and library preparation

Genomic DNA extraction from 1.8 mL of sample (homogenized kefir grains and fermentation liquor) was performed using the DNeasy Ultraclean Microbial Kit (QIAGEN, Hilden, Germany) with the following modifications: treatment of the kefir grains with 0.9% saline solution prior to extraction and two additional steps to improve the cell lysis consisting of lysozyme incubation (37°C) for 15 min and water bath (70 °C) for 10 min + enzyme treatment with Proteinase K (QIAGEN, 2020). Extracted DNA for each sample was verified for its quality and integrity through electrophoresis gel and nanodrop quantification. Genomic samples were used to construct multi-amplicon libraries using the SWIFT AMPLICON^®^ 16S + ITS PANEL protocol (Swift Biosciences, Ann Arbor, United States) with a primers pool covering all variable regions of the 16S rRNA gene (V1-V9) and the fungal ITS 1 and ITS 2 genes for the identification of bacteria and yeast (Swift Biosciences, 2018; Gao and Zhang, 2019). The sequencing process was performed using the Iseq 100 system with a 2 × 150 bp read length (Illumina, United States) at EAFIT University sequencing facility (AXOMICS).

### 2.5 Metabarcoding sequencing and data preprocessing

Sequence data processing was subjected to quality checks and analyzed using Qiime 2 workflow (Bolyen et al., 2019). For the bacterial analysis, obtained reads were divided by each region (V1–V9 of the 16S rRNA gene), and reads from the V4 region were selected to arrange the data into Amplicon Sequence Variants (ASVs) using DADA2, also correcting errors in sequences by removing singletons, chimeric sequences, and dereplicating data (Callahan et al., 2016). Data was then clustered into OTUs (Operational Taxonomic Units) with a 97% similarity. The taxonomic classifier for the classification was SILVA 138 SSU and GTDB databases for bacterial reads and UNITE for yeast identification (Beccati et al., 2017; Kõljalg et al., 2020; Parks et al., 2020). After quality checks and taxonomic categorization were done, alfa and beta diversity metrics were determined using the Phyloseq package and the integrated development environment for R, RStudio (version 1.4.1106) (RStudio Team, 2020).

### 2.6 Untargeted metabolomics analysis

As stated in Figure 1, samples were collected from a second curve at five different fermentation times (0, 72, 82, 92, and 120 h) and one media control (molasses) was also included. These samples were stored at −80°C until metabolite extraction was performed. Each fermentation time had four replicates resulting in a complete dataset of 24 samples to be analyzed. Liquor and control extracts were obtained by methanolic extraction using 50% MeOH. Briefly, 20 mL of each homogenized sample was mixed with 4X solvent in sterile glass flasks; afterward, the solution was sonicated at 30% amplitude for 20 min (pulse function 1 min on + 30 s off) and agitated in the dark at 200 rpm for 4 h. The resulting solution was centrifuged at maximum speed (4,500 rpm) for 15 min and filtered with a vacuum pump and Whatman filter papers (grade 2). Solvent evaporation was performed with a Rotavapor R-300 using the manufacturer’s instructions (BÜCHI, Flawil, Switzerland). Extracts were vacuum evaporated with the Concentrator Plus system (Eppendorf; Hamburg, Germany). The final solid residue was Weighed and resuspended in 2 mL of 50% MeOH for downstream procedures.

### 2.7 Metabolomic data acquisition and pre-processing

The resulting 24 extracts (4 replicates per sample) were sent to the Metabolomic Core Facility (MetCore) at the Universidad de los Andes in Bogotá-Colombia. Samples were vortexed at 3,200 rpm and filtered on 0.22 µm filters. Then, 80 µL of each extract was taken for subsequent untargeted metabolomic analysis using an Agilent Technologies 1260 Liquid Chromatography system coupled to a 6545 Q-TOF quadrupole time-of-flight mass analyzer with electrospray ionization (RP-LC/MS-QTOF). For the reverse phase, the injection volume of the samples was 10 uL and the compound separation was done on a C18 column (InfinityLab Poroshell 100 × 3.0 mm, 2.7 μm) at 40°C. The mobile phases used for elution were composed of 0.1% (v/v) formic acid in Milli-Q water (Phase A) and 0.1% (v/v) formic acid in acetonitrile (Phase B) pumped at 0.4 mL/min. The mass detection was performed in positive ESI mode on autoMS/MS from 50 to 2000 m/z. Tandem mass spectrometry data obtained was pretreated and converted to mzML format using MSConvert GUI (Adusumilli and Mallick, 2017). Throughout the analysis, two reference masses were used for mass correction: *m/z* 121.0509 (C_5_H_4_N_4_), and *m/z* 922.0098 (C_18_H_18_O_6_N_3_P_3_F_24_) corresponding to protonated purine and protonated hexakis, respectively.

### 2.8 Molecular networking and metabolites identification

mzML converted files were uploaded to the GNPS server using an FTP client with the corresponding input parameters. Then, the files (ftp://massive.ucsd.edu/MSV000091955/) were used to perform Classical Molecular Networking) in the same platform (Wang et al., 2016), and the resulting networks were visualized on Cytoscape version 3.9.1 (Shannon et al., 2003). Different analyses were run by changing some of the network parameters, including min pairs cosine value, min of fragment ions, and number of matched peaks with final parameters stated in Table 1. After these analyses, the molecular network that best suited the objective (broad identification with rigor of a minimum of six peaks identified) was selected to perform manual curation and annotation of the chemical families. The resulting feature table with all nodes’ information was downloaded in “.csv” format from Cytoscape version 3.9.1. Then, each subnetwork information was downloaded and treated as a cluster or chemical family. Each annotation or library identification within a subnetwork was revised for its level of identification (either gold or bronze and number of shared peaks) with reference to the spectra and peak list downloaded from public databases (MassBank of North America—MoNA (https://mona.fiehnlab.ucdavis.edu/) and CEU Mass Mediator (Gil-De-La-Fuente et al., 2019). In this way, when a single node or feature was annotated within a cluster (subnetwork), all neighbor nodes were grouped and named under the chemical family of this annotated compound, since the clustering algorithm groups by structural similarity according to the parameters set for the network (Table 1) (Schrimpe-Rutledge et al., 2016). Thus, each cluster was named when a single or multiple features in it were correctly annotated by library detection in GNPS, and the reference MS/MS of the compound was checked from public repositories using MassBank of North America—MoNA (https://mona.fiehnlab.ucdavis.edu/) and CEU Mass Mediator (Gil-De-La-Fuente et al., 2019). This procedure was specifically done for nodes denoting a weak annotation (six shared peaks and bronze standard). For the nodes that were not identified by GNPS libraries and with the aim of expanding the networks’ annotation, a manual search of selected features was done using the precursor mass value as a query in chemical databases, namely, PubChem (Kim et al., 2021) ChemSpider (Pence and Williams, 2010), the Atlas of Natural Products (Santen et al., 2022). Each precursor mass on each node was also checked to unveil the presence of any of the three most common adducts, e.g [M + H+]. If a hit was found, the reference MS/MS was compared with the network feature, and if more than six peaks were found in common, an annotation hit was called. As an alternative way of propagating annotation of the network, if a cluster had more than one identified node, the chemical family that grouped all the annotations of the cluster was selected to name the cluster (e.g., haematommic acid and L-beta-3-phenyllactic acid were identified in the same cluster, both with a benzene ring in the structure and belonging to the more general category of phenolic acids). Finally, the modified network with the annotated notes was downloaded in a high-resolution image format (“.png” with 600 DPI), with the annotated chemical families highlighted in circles and named after each search.

**TABLE 1:** Parameters used for Classical Molecular Networking in GNPS platform.

Parameters	Values
Minimum pairs cosine	0,7
Minimum fragmented ions	6
Cluster size	2
Minimum matched peaks	6
Search analogs	Do search

### 2.9 Statistical analysis using metaboanalyst 5.0

Simultaneously to the molecular networks, raw data obtained from RP-LC/MS-QTOF was converted to mzML format and uploaded to the Metaboanalyst 5.0 (https://www.metaboanalyst.ca/) (Pang et al., 2022). This platform was used to perform multivariate analyses on all samples from the ‘LC-MS Spectra Processing’ option. Principal components analysis (PCA) was performed using the default parameters in order to validate the significance of the sampling and to obtain insights about the treatments used in the experiment (fermentation times).

### 2.10 Statistical and integrative analyses

After the analyses were performed, statistical methods were applied to visualize the distribution patterns between the two types of samples (kefir grains and liquor) and the fermentation times using the integrated development environment for R, RStudio (version 1.4.1106) (Rstudio Team, 2020). Filtered data from the compositional metagenomic approach consist of ASVs that had more than 32 sequencing reads. Then, dominant species ASVs were selected to perform statistical validation through a non-parametric Mann—Whitney *U* test (*p*-value <0.05) using the function wilcox. test() in the R Stats package, looking for significant differences between the treatments (fermentation times and sample origin) (Supplementary Additional file S4) (Rstudio Team, 2020). In addition, the Pearson correlation test was conducted as a primary approximation to the association between observed ASV and annotated chemical families through the R function cor() from the R Corrplot package (Wei and Simko, 2021).

## 3 Results

### 3.1 Culturable microbial communities in water kefir

**FIGURE 2:**
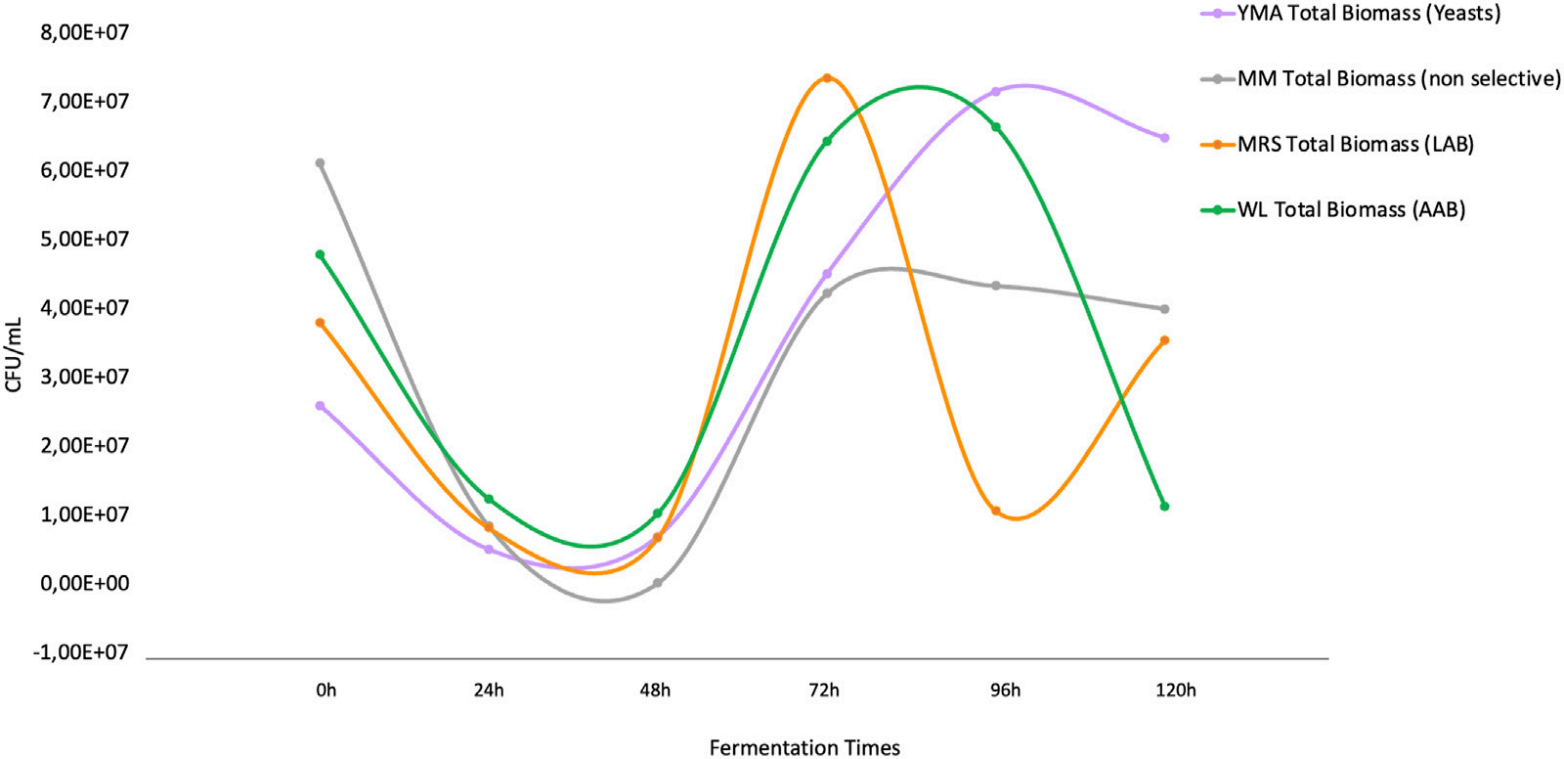
Population dynamics based on **culture-dependent methods** of the four microbial groups in the study. The orange line indicates the growth of lactic acid bacteria (MRS media), the green line indicates acetic-acid bacteria (WL media), the purple line indicates yeasts (YM media), and the gray line indicates total molasses degrading biomass (MM media).

The dynamics of representative microbial groups cultured from the fermentation are shown in Figure 2. It was observed that the groups under study had a relatively similar growth pattern during the fermentation process until 72 h post-inoculation of the grains was reached. Molasses culture media (MM) reflects an average of the culturable biomass, non-biased to specific nutritional compositions of the other media but supporting the growth of the entire community in the fermentation. The total biomass in the media was the lowest after 72 h (4.22E+07 CFU/mL), reflecting a stationary phase with a slight negative slope. Instead, for the other culture media that were selective for specific microbial groups (See Materials and Methods), high microbial growth was observed at 72 h for *Lactobacillus* sp. communities that were selectively grown in MRS media (Figure 2), which decreased rapidly in nearly 1 order of magnitude and retook a second stage of growth after 96 h. For acetic acid bacteria growing selectively in WL medium, the peak growth was observed before 72 h and lasted until after 96 h of fermentation, then rapidly decreasing and alternating with the regrowth of *Lactobacillus* sp. For yeasts growing in YM agar media, the peak growth is observed at the end of the fermentation, at exactly 96 h, after which, it starts to decline.

### 3.2 The strains collection includes the most representative groups reported for water kefir, exhibiting probiotic potential

A total of 95 samples were isolated, 73.7% represented by bacteria and 26.3% by yeasts. In addition, they were classified by the source of isolation resulting in 63.12% of the strains coming from fermentation liquor and 29.47% from kefir grains (Table 1, Supplementary Additional file S1). Among the microorganisms identified by Sanger sequencing, we could identify *Lentilactobacillus hilgardii, Lactobacillus buchneri, Lacticaseibacillus paracasei, Schleiferilactobacillus harbinensis, Acetobacter pasteurianu*s, and *A. tropicalis* (Gulitz et al., 2011; Olivo et al., 2017; Lynch et al., 2021; Rodríguez et al., 2022) and yeasts such as *Pichia membranifaciens* and *Kazachstania exigua* (Nejati et al., 2020; Moretti et al., 2022). These microorganisms have been previously reported to have probiotic effects and, thus, provide a useful resource for the underlying interest of the project in providing a knowledge and resource basis for the future development of health-beneficial microbially derived supplements, such as probiotics and postbiotics.

### 3.3 Total microbial communities obtained through compositional metagenomics reflect similar patterns to what is observed in culturable population dynamics and suggest an enrichment of species at 72 h of fermentation

**FIGURE 3:**
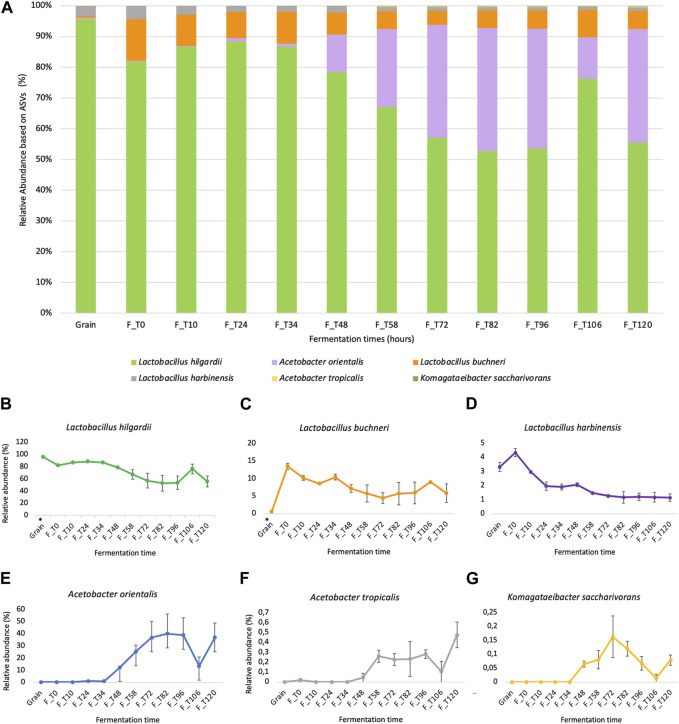
Relative abundance of dominant bacterial ASV identified through compositional metagenomics analysis. **(A)** Relative abundance of the six dominant bacterial ASVs on each fermentation time sample between 0 h and 120 h, including the grain inoculum. **(B–G)** Ind Individual kinetics showing the behavior of each identified ASV over time (*L. hilgardii*, *L. buchneri*, *L. harbinensis, A. orientalis*, *A. tropicalis,* and *K. saccharivorans*). The (*) in grain samples for *L. hilgardii* and *L. buchneri* indicates statistically significant differences in the abundance values for these groups according to sample origin (Mann-Whitney *U* test, *p*-value = 0.007246).

The successful sequencing of the prepared libraries derived from the grains inoculum (2) and fermentation liquor (22) resulted in a dataset of 24 samples with a combined size of approximately 6.23 Gbp and a quality score (Q30) of 94.48% (Supplementary Table S2, Supplementary Additional file S1). The obtained results from compositional metagenomics (V4 region of the 16S rRNA gene) support the hypothesis that the microbial consortium corresponds to a water kefir-derived product since the main groups belong to lactic acid bacteria, acetic acid bacteria, and yeasts (Fiorda et al., 2017; Çevik et al., 2019; Guzel-Seydim et al., 2021; Patel et al., 2022; Yerlikaya et al., 2022). According to the relative abundances of the six dominant species that can be observed in Figure 3, it was possible to observe that *L. hilgardii* was dominant in the grain sample, representing ∼90% of it, and at early fermentation times until 34 h, starting to decrease after 48 h of the process to ∼50% representation. For acetic acid bacteria instead, they increased from 1%–12%–∼40% between 34 and 48 h of fermentation.

The kefir grains that were used as fermentation inoculum were mostly represented by lactic acid bacteria, which is consistent with previous studies in sugary beverages (Yerlikaya et al., 2022) (Supplementary Additional file S2). The results are partially in accordance with the observations in the population dynamics of culturable microbial groups (Figure 2); other species, mainly *Acetobacter orientalis* and *Lactobacillus buchneri*, unlike those in the grain (mainly *L. hilgardii*), increased significantly after 72 h, which can be explained from the perspective of the multispecies interactions and metabolite production taking place in the system. For the case of *L. hilgardii* and *L. buchneri,* statistically significant differences were found between the grains and fermentation liquor (*p-*value *=* 0.007246) through the Mann-Whitney *U* test. For the members of the family Acetobacteraceae, no significant test were performed since this group was not detected on the grain inoculum, but it is obvious that the population change is significant, passing from almost non detectable to representing ∼40% of the microbial community in the matrix. Regarding the metabarcoding results for the ITS region (ITS1-ITS2), all obtained reads were classified as *P. membranifaciens*, given its high abundance. Besides, there is evidence of coverage of the panel used for the metataxonomic libraries preparation favoring the 16S rRNA gene regions for bacterial identification (Figure 1, Supplementary Additional file S1).

### 3.4 Biodiversity of the water kefir fermentation decreases in the first stages of fermentation and restarts again after 48 h post-inoculum

**FIGURE 4:**
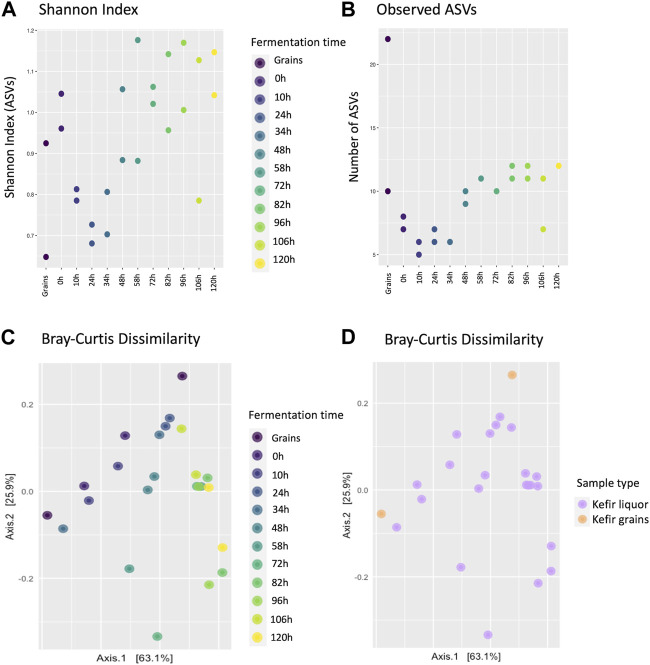
Estimation of diversity indices of the microbial communities during the fermentation process, based on the normalized ASVs counts/ per volume of sample for the microbial communities identified during the fermentation process. **(A)** Scatter plot showing the Shannon index (alpha diversity) estimated for each fermentation time, according to the identification of ASVs and grouped by fermentation sample. **(B)** Scatter plot of the number of identified ASVs on each sample classified by fermentation sample. **(C)** Scatterplot for the Bray-Curtis dissimilarity index (beta diversity) for classified ASVs on the fermentation liquor samples and grains inoculum. **(D)** Scatterplot for the Bray-Curtis dissimilarity index estimated by fermentation sample type.

The biodiversity of the grains inoculum and fermentation liquors was measured in terms of ecological indices using the Phyloseq package from the integrated development environment for R, RStudio (version 1.4.1106) (Supplementary Additional file S2). Alpha diversity was measured as the richness or dominance of species in the microbial communities of each sample. Shannon index values between 0.7 and 1.2 were obtained (Figure 4A). These diversity values indicated a gradual decrease in diversity between 10 and 34 h, which increased again after 48 h of fermentation.

In the same way, it was observed that the number of identified ASVs presents differences according to each fermentation time (Figure 4B). This result coincides with previous studies that show that the grain and liquor environment differ in microbial composition, with the grains typically having a higher microbial load (Moretti et al., 2022; Patel et al., 2022). Regarding the replicates, differences were observed between the values of the Shannon index and the number of ASVs for some of the replicates at each time, specifically in the grain inoculum and fermentation liquor at 48, 58, 82, 96, and 106 h.

In terms of b-diversity, the Bray-Curtis dissimilarity index was calculated and visualized by each fermentation time and the sample type. There was not a clear clustering based on the fermentation time; however, it was possible to observe a “vanishing” pattern from the top to the bottom of the graph (Figure 4C). According to the sample type, it was found that the two grain samples differed from each other, and for the fermentation liquor, there was no clustering pattern (Figure 4D).

### 3.5 Chemical space of the biological system composed by the water kefir community clusters in two groups according to multivariate statistical analysis

**FIGURE 5:**
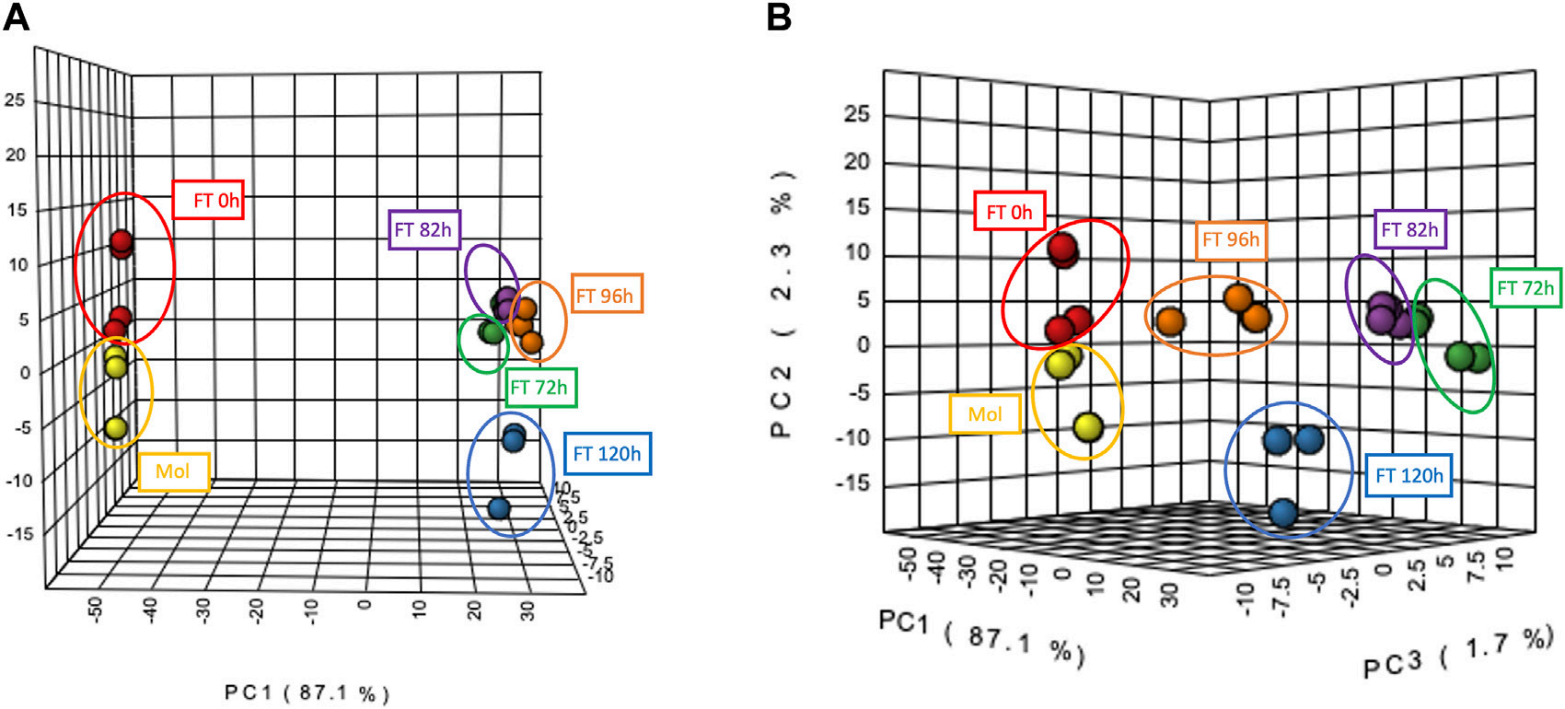
Principal Components Analysis (PCA) among molasses and fermentation samples generated by Metaboanalyst 5.0. **(A)** Red and Yellow clusters represent samples from molasses and fermentation time 0 h; green, purple, blue and orange clusters represent samples from the latest fermentation times from 72 to 120 h. **(B)** Distribution of the four replicates for each of the six fermentation samples.

In order to analyze the system from a chemical point of view, a multivariate statistical analysis using Metaboanalyst was performed and allowed to group the samples by their chemical composition in two clusters based on the principal component 1 (PC1), suggesting that samples corresponding to molasses and early stages of fermentation exhibit a similar metabolite profile among them, while the samples from the latest times (72–120 h) of the fermentation share a common chemical profile (Figure 5A). This clustering was consistent with enrichment points identified by previous methods, indicating microbial interactions that could lead to the release of different amounts of metabolites. In addition, the four replicates of each treatment (fermentation time or control/molasses) were able to cluster together. This indicates that there are no significant differences within each replicate of a treatment between fermentation times (Figure 5B).

### 3.6 Untargeted metabolomics evidences a rich chemical biology derived from the water kefir fermentation, with different reported bioactivities

**FIGURE 6:**
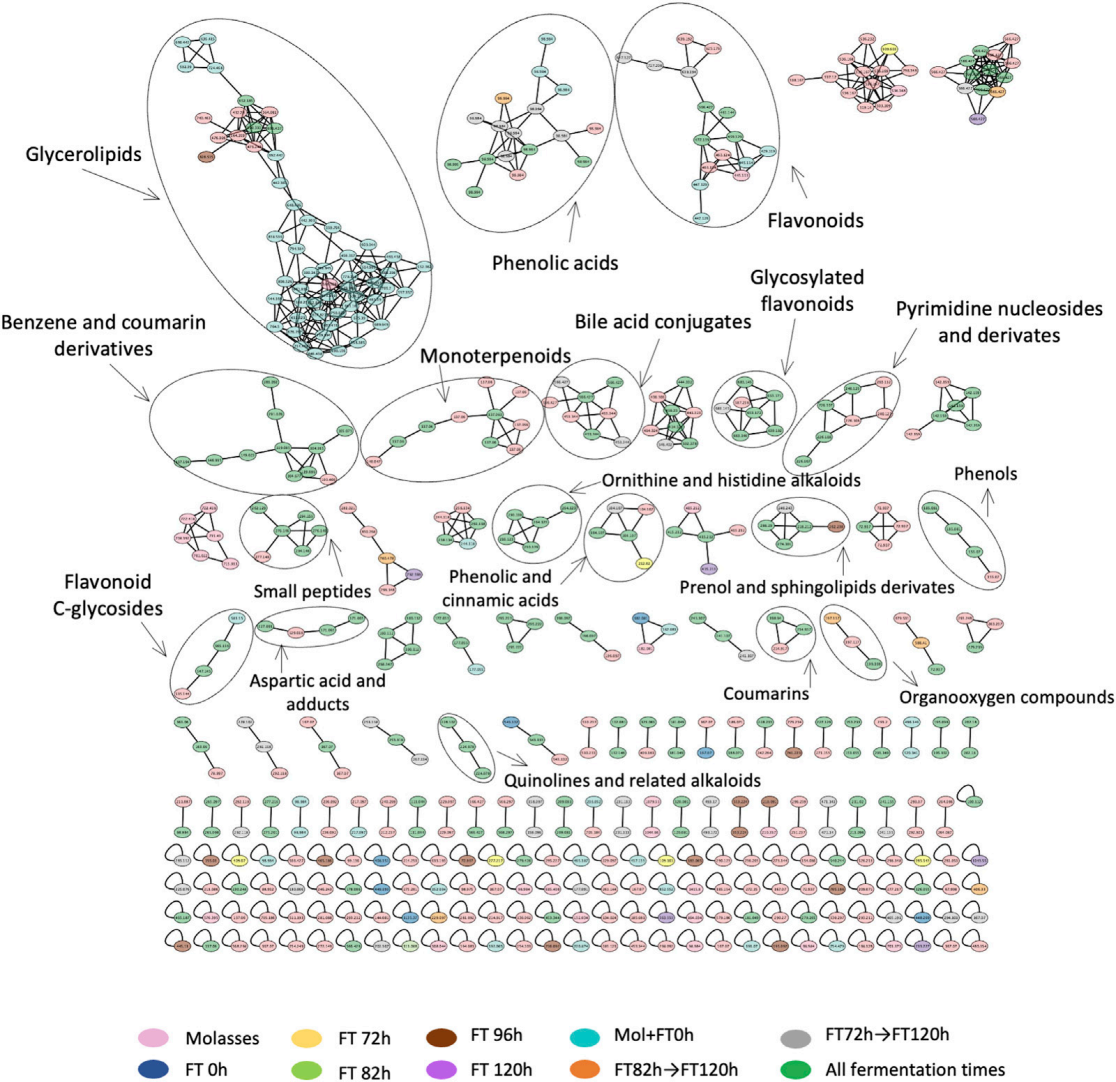
Curated molecular network with identified chemical families from the extracts processed by RP-LC/MS-QTOF. Nodes are colored by treatments: molasses control (pink), 0 h liquor (dark blue), 72 h liquor (yellow), 82 h liquor (green), 96 h liquor (brown), 120 h liquor (purple), molasses +0 h liquor (cyan), from 82 h to 120 h (orange), from 72 h to 120 h (gray), and presence in all groups (dark green).

757 nodes and 260 compound annotations were obtained from the molecular network generated by GNPS bioinformatic platform (https://gnps.ucsd.edu/ProteoSAFe/status.jsp?task=2c80ff4b1e2a49fea7e844644198bbf3) with the selected criteria (Table 1). After manual curation and annotation, it was possible to identify 18 chemical families, such as phenolic acids, quinolines, flavonoids, monoterpenoids, organic acids such as lactic acid, and amino acids (Figure 6, Supplementary Additional file S3 Spectra_confirmation). It was possible to highlight the fermentation sample where each family was prevalent based on the number of spectra identified in each sample. At the beginning of the fermentation (Molasses and FT 0 h), the chemical families identified were glycerolipids and monoterpenoids. Families such as phenolic acids, flavonoids, and benzene products were found during the latest times (between 72 and 120 h).

### 3.7 Bacterial group presence in specific times of water kefir fermentation correlates with the enrichment of chemical families identified through metabolomics

**FIGURE 7:**
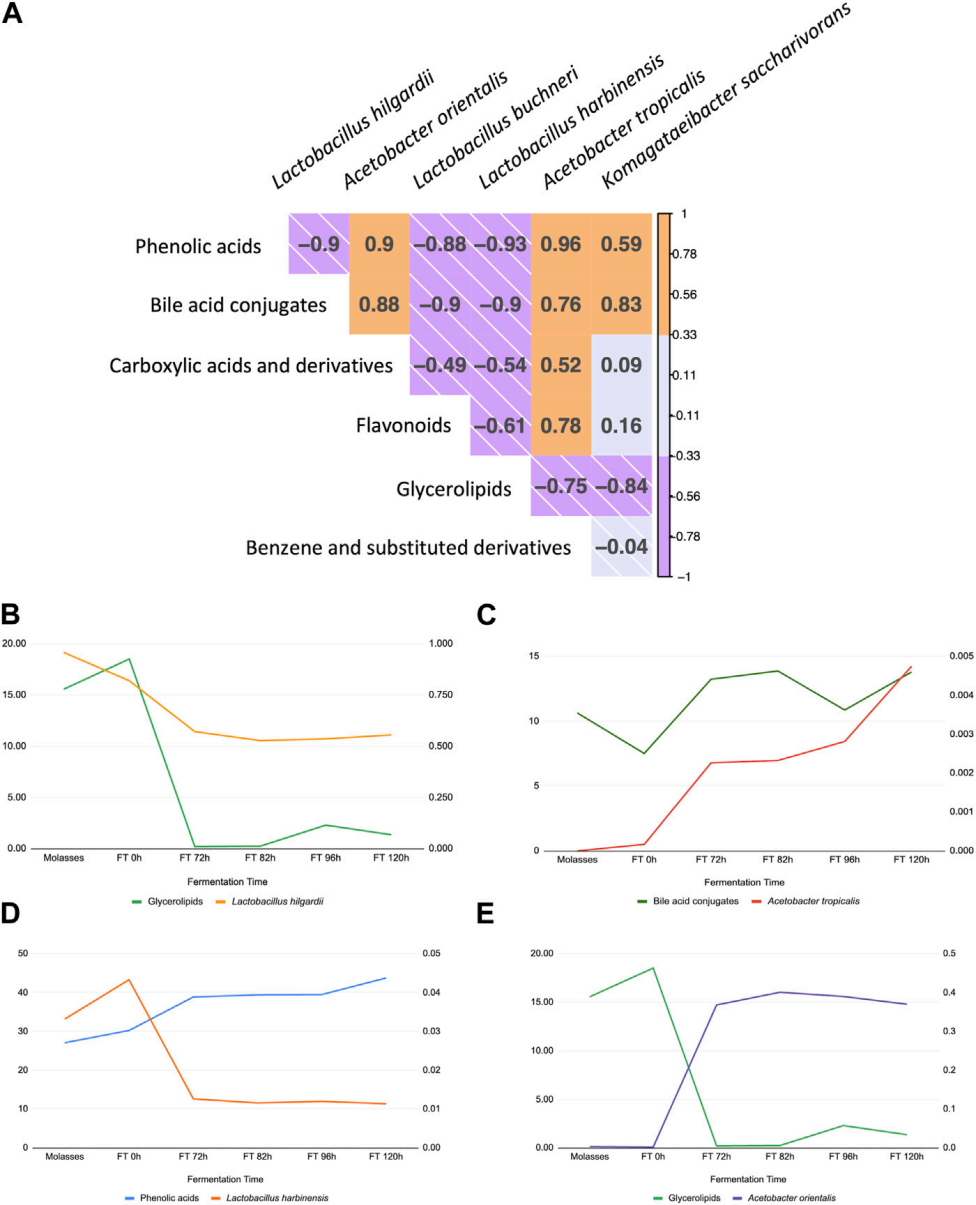
Correlation between compositional metagenomics and identified chemical families during the fermentation of the microbial consortia. **(A)** Correlation visualization between dominant ASVs, three *Lactobacillus,* and three *Acetobacter* taxa with dominant chemical families reported from GNPS molecular network. **(B–E)** Individual plots showing the change in each metabolite and taxa of interest over time (*L. hilgardii, L. harbinensis, A. orientalis,* and *A. tropicalis*), supporting the association obtained by Pearson’s correlation.

The correlation was estimated between the six dominant ASVs identified down to the species level and the six dominant chemical families from the fermentation extracts (Figure 7). *Lactobacillus hilgardii*, *L. buchneri,* and *L. harbinensis* decrease in a direct correlation with glycerolipidss, a chemical family of compounds that plays important roles in cell signaling, membrane trafficking, and anchoring of membrane proteins (Henry et al., 2012). *Acetobacter orientalis* and *A. tropicalis* were positively correlated with four out of six chemical families. Furthermore, some metabolites of interest (e.g., phenolic acids) were plotted individually with observed dominant ASVs (Figures 7B–E), finding that *A. tropicalis* and bile acids conjugate correlate directly (Figure 7C), while *L. hilgardii* and glycerolipids have a direct correlation more pronounced at later stages of the fermentation, while *A. tropicalis* and the same chemical family have a negative correlation (Figures 7A, D, respectively), suggesting the sensibility of this species for these compounds or a capacity for degradation. Furthermore, phenolic acid concentration inversely correlates with populations of *L. harbinensis*.

## 4 Discussion

The use of different ‘omics’ techniques to study fermented matrices has significantly increased during the last decade, given the broader scope they offer to gain an understanding of the different layers involved in any biological system. Nevertheless, no reports of the integration of culture-dependent methods with compositional metagenomics have been found prior to this research, making this the first of its kind. According to the findings of the study, the WK fermentation of the study, including its modifications according to local Colombian culture, still corresponds to a water kefir-derived product. The before is stated, since the main microbial groups found belong to lactic acid bacteria, acetic acid bacteria, and yeasts, as is expected from this kind of biological system (Fiorda et al., 2017; Çevik et al., 2019; Guzel-Seydim et al., 2021; Patel et al., 2022; Yerlikaya et al., 2022). The majority of reports on the composition of water kefir report *Lactobacillus* and *Acetobacter* as the representative genera in these fermentation processes (Gulitz et al., 2011; Fiorda et al., 2017; Verce et al., 2019). Some previous studies have also described dominant bacteria of the genera *Streptococcus*, *Leuconostoc,* and *Lactococcus* and yeast genera such as *Dekkera* and *Saccharomyces* (Laureys et al., 2022; Pihurov et al., 2023). Contrary to this last report, we found in our WK a hybrid matrix with a major presence of *Lactobacillus* sp. and *Acetobacter* sp but an absence of *Lactococcus*, *Leuconostoc, and Streptococcus* sp. on the bacterial composition. Regarding yeasts, we found a high abundance of *P. membranifaciens, Pichia kudriavzevii,* and *K. exigua.* This finding is interesting since some studies, which even change substrate conditions (Laureys and de Vuyst, 2016; Zannini et al., 2023), have not detected *P. membranifaciens* nor *K*. *exigua*, denoting the uniqueness of this WK fermentation and its chemical space. Specifically, this species, *K. exigua,* has been more commonly found in olive brine, wine, and other fermented matrices (Jood, I et al., 2017). There have also been some reports of its presence in superficial and subterraneous waters, sediments, and soils in crude extracting zones (SIB Colombia, 2023). According to the relative abundances of dominant species (Figure 3), *L. hilgardii* was observed to be dominant in the grain sample and during early fermentation times, starting to decrease after 48 h of the fermentation, which is probably related to the proliferation of other community members that compete for substrate and generate a novel chemical environment. Furthermore, this species has been associated with water kefir fermentations and is thought to be responsible for the growth of kefir grains due to the production of EPS, such as dextran, which is a sucrose derivate with great potential in the industry due to its relative stability and good solubility (Lebeaux et al., 2014; Laureys and de Vuyst, 2016; Lynch et al., 2021). Lactic acid bacteria are a commonly known group due to their probiotic effects and the production of organic acids that preserve and improve the aromatic and bioactive qualities of water kefir and other fermented foods (Azi et al., 2021; Spizzirri et al., 2023). Even though we found a high abundance of various *Lactobacillus* species among the most abundant microbial groups, several other species of acetic acid bacteria and some yeasts increased after 72 h, suggesting that multispecies interactions and metabolite production contributed to their growth. These observations could be explained by the associations between different microorganisms, e.g., the acidification of the media by *Lactobacillus* species and the use of end-products as an energy source can improve the growth of acetic acid bacteria and yeasts; meanwhile, essential nutrients released by yeasts can support bacterial growth (Pendón et al., 2021). Regarding the fermentation substrate, molasses has been previously reported as a low-cost alternative for industrial fermentation of water kefir with a variety of potential biological activities, such as antioxidant capacity, due to the presence of sugar-derived compounds that induce the growth of microorganisms of interest as well as the production of metabolites that facilitate multispecies interactions (Deseo et al., 2020; Mordenti et al., 2021).

As we stated before, the relative number of reads for lactic acid bacteria decreased in time, and we observed that acetic acid bacteria species appeared in a relatively high abundance after 34 h, which could be due to their ability to grow in low concentrations of oxygen and, at the beginning of the fermentation, there is high oxygen availability and higher sugar levels in the media (Pendón et al., 2021) (Figures 3E, G). The genus *Acetobacter* has been reported in the microbial communities of kefir-based fermentations, but its role has not been fully elucidated, except for the fact that they can contribute to the flavor and aroma of the final fermented product and metabolize sugar and alcohol through the pentose phosphate pathway to accumulate large amounts of diverse fermentative products including D-sorbitol, ascorbic acid, and the prebiotic levan, which are derived from the principal metabolite of this group, acetic acid (Azi et al., 2021; Patel et al., 2022; Yerlikaya et al., 2022). *Acetobacter orientalis* and *A. tropicalis* have been reported as the dominant species in previous studies. These species are known as fermentation stabilizers and contributors to the aroma of water kefir (Gulitz et al., 2011; Martínez-Torres et al., 2017; Guzel-Seydim et al., 2021). We corroborate these findings, with the two most prominent acid bacteria species being *A. orientalis* and *A. tropicalis* but correlating their appearance in the fermentation with phenolic acids of several classes and molecular weights, with monoglycerides, isoleucine derivatives, and flavones (Figures 6, 7). Also, an interesting proportion of the acetic acid bacteria *Komagataeibacter saccharivorans* was found in this system. This one is a producer of cellulose and has been mostly found in milk kefir but not in water kefir grains.

On the other side, regarding biodiversity measures, low diversity values for index at the start of the fermentation could be explained due to the dominant microorganisms found in the matrix that belong to a few genera, such as *Lactobacillus* and *Acetobacter*. These diversity values indicate a gradual decrease in diversity between 10 and 34 h, which increased again after 48 h of fermentation. This behavior was associated with previous studies, such as that of Patel et al. (2022) and their diversity analysis in kefir grains and liquid from the sequencing of the V3-V4 region of the 16S rRNA gene. Similarly, it was observed that the number of identified ASVs presented differences according to the fermentation sample. This result coincides with previous studies in which it has already been established that the grain and liquor environments differ in microbial composition, with the grains typically having a higher microbial load (Moretti et al., 2022; Patel et al., 2022). This result could be explained since the kefir grains are the main source of microorganisms, and biodiversity in the beverage can vary based on the substrate, culture conditions, and microbial interactions that occur during the fermentation (Fiorda et al., 2017; Pendón et al., 2021; Moretti et al., 2022). Apparent differences between replicates were also observed, which could be related to population dynamics and/or sample manipulation, considering that they differ more in the value of diversity and not in the number of taxa identified (e.g., grain replicates). In terms of b-diversity, the results suggest that at the beginning of fermentation, there are more different species, while at the end of the process, the samples share a greater number of species. Furthermore, no clustering was observed for fermentation liquor samples. This can be explained by the fact that the microbial consortium is a complex and dynamic biological system that can exhibit different characteristics under similar conditions.

The results of metabolite screening were consistent with previous targeted studies designed to look for organic acids, alcohol levels, produced EPS, and other compounds derived from the fermentation process of water kefir grains (Plessas et al., 2017; Azizi et al., 2021; Moretti et al., 2022; Esatbeyoglu et al., 2023). From the identified metabolites, it was observed that there could be a correlation between the microorganisms found and the biological potential of the fermented product. For example, inhibitory effects against pathogens have been associated with the production of protective organic acids by lactic acid bacteria and ethanol produced by yeasts (Yerlikaya, 2019). The formation of organic acids released by LAB species in water kefir is an important indicator of enhanced metabolic activity since they may be used as a substrate by other groups of microorganisms (Bulat and Ali, 2021; Satir, 2022). Previous studies have shown that ß-glycoside enzyme and phenolic acids (e.g., haematommic acid and lactic acid) produced during the microbial fermentation release glycosylated or bound flavonoids such as saponarin and puerarin and produce new polyphenols (Azi et al., 2021). Furthermore, water kefir has a high antioxidant potential due to the phenolic compounds and enzymes found as fermentation end-products (Cai et al., 2020; Yerlikaya et al., 2022). Most of the bioactive components are classified as polyphenolic compounds in nature, which include the identified cinnamic acid, coumarin, thymol, and myrcene (Satir, 2022). In the generated molecular network, it was found that this chemical family was dominant in the latest times of fermentation, suggesting its relationship with acetic acid bacteria metabolism and other multispecies interactions. In addition to their role against oxidative stress, some phenolics have been reported as antimicrobial agents, including benzoic acid, which was also annotated in the molecular network (Rodrigues et al., 2016; Azi et al., 2022). In this way, the increase in the total phenolic compounds is strongly correlated with an increase in antioxidant activity, which is also associated with an anti-inflammatory effect of water kefir fermentations (Rodrigues et al., 2016). From the identified metabolites, it was possible to annotate and validate by reference spectra a compound of interest that had not been reported for water kefir-based fermentations. Isoschaftoside is a C-glycosyl flavonoid originally extracted from root exudates of *Abrus cantoniensis* (Guan et al., 2022). Generally, C-glycosyl flavonoids are part of the diet and have been reported to have a wide range of pharmacological activities including blood-lipid-lowering, hypoglycemia, neuroprotective, antitumor, and antioxidant capacities (Tremmel et al., 2021; Guan et al., 2022). The C-glycosylation of flavonoids gives rise to more stable, biologically active metabolites with different spectral properties and increased solubility in polar media compared to O-glycosides (Brazier-Hicks et al., 2009; Vanegas et al., 2018; Khodzhaieva et al., 2021).

From the resulting molecular network, an estimate of the relative abundances for each chemical family was obtained based on the number of spectra found on each sample. The dominant chemical family among the fermentation was that of phenolic acids, with increasing values after 72 h, which suggests their association with microbial interactions. As mentioned above, phenolic acids are known as bioactive compounds with a wide range of applications and have been associated as end-products from lactic-acid bacteria metabolism, which could explain their relative high abundance since the beginning of the fermentation, where lactic-acid bacteria are more abundant and increase in the latest times, possibly related to the presence of yeast and acetic acid bacteria (Păcularu-Burada et al., 2022).

Finally, correlation primary analyses suggested that *Lactobacillus hilgardii*, *L. buchneri,* and *L. harbinensis* showed a positive correlation with glycerolipids, a chemical family of compounds that play important roles in cell signaling, membrane trafficking, and anchoring of membrane proteins (Henry et al., 2012), an association that could be explained by their decrease in relative abundance over the fermentation course and the changes in the fermentation conditions (e.g., pH values, oxygen levels, multispecies interactions), which could also support its negative correlation with *Acetobacter*, representing microorganisms that are tolerant to these environmental conditions or that can degrade this family of compounds. *Acetobacter orientalis* and *A. tropicalis* were reported to be positively correlated with five out of six chemical families, including phenolic acids of several classes and molecular weights, with monoglycerides, isoleucine derivatives, and flavones, which support the hypothesis that the latest fermentation times represent key stages for bioactive compound detection and the production of interesting molecules from the therapeutic and functional nutrition point of view (Figure 7). These findings altogether, namely, the integration of metataxonomic data with the enrichment of chemical families, and the identification of representative strains isolated from this biological system (water kefir fermentation) represent a straightforward approach to unlocking the potential of fermented foods with proven health benefits, taking a step further toward the design of targeted formulations based in microorganisms and their metabolites (probiotics or live microbial biotherapeutics, postbiotics). Strategies like the one we propose in this research can translate the benefits of fermented foods beyond their prescription to be consumed daily into active pharmaceutical products or active ingredients since most of them do not naturally reach the required concentrations to be considered therapeutic or efficient, but the rational design of a product based on their properties and their microbial strains can be harnessed using bioprocesses and biotechnology to achieve this status.

## 5 Conclusion

Functional foods are gaining interest due to the increase in non-communicable diseases like diabetes, obesity, and cardio-metabolic conditions. The results presented in this work correspond to a primary study developed on a water kefir product, a type of fermented food locally produced in Colombia, with modifications to its traditional way of preparation. The results of culture-dependent and molecular methods showed consistent findings in microbial richness and metabolite production increasing between 72–96 h, with dominant microorganisms identified as *L. hilgardii* (LAB), *A. orientalis* (AAB), and *P. membranifaciens* (yeast). Untargeted metabolomics using molecular networking allowed the generation of hypotheses on which small molecules are being produced during fermentation. Phenolic acids, flavonoids, and monoterpenoids are of great interest given the reported health benefits represented in the most abundant chemical families, which are produced to a greater extent by bacteria, namely, by *Acetobacter* species, specifically *L. hilgardii* for beneficial lipids and *A. tropicalis* for phenolic compounds and bile acid conjugates. In addition, we annotated and subsequently confirmed by reference spectra the presence of Isoschaftosides, a group of compounds that can be promising for the development of products derived from this microbial consortium. This study is the first of its kind in a fermented water kefir matrix locally produced in Colombia and the first to report the enrichment of chemical families, such as Isoschaftosides and other flavonoids produced by fermentation of a WK-derived microbial consortium, This study, despite being a first approximation of the chemical space of a Colombian-based WK fermentation and its correlation to the microbial taxa involved, provides a solid basis for future studies elucidating the mechanism of action of these functional fermented foods by directed metabolomics or analytical chemistry or on the isolation of chemical compounds of nutritional and therapeutic interest from fermented food matrices. We conclude that this study contributes to the existing knowledge on the dynamics of kefir fermentation and highlights the unique biological potential that each version can exhibit, as well as providing specific knowledge that could be easily applied to the rational development of novel probiotic and postbiotic ingredients for functional nutrition.

## Data Availability

The datasets presented in this study can be found in online repositories. The names of the repository/repositories and accession number(s) can be found in the article/Supplementary Material.
