# Supplementary material for: Multi-omics characterization of the microbial populations and chemical space composition of a water kefir fermentation
Source: Front Mol Biosci. 2023 Oct 2;10:1223863. doi: 10.3389/fmolb.2023.1223863 (PMC10577418; doi:10.3389/fmolb.2023.1223863)
Supplement: Supplementary file 2 [file DataSheet1.PDF]

Additional File 1

MULTI-OMICS CHARACTERIZATION OF THE MICROBIAL AND CHEMICAL ECOLOGY OF A WATER KEFIR FERMENTATION

Maria Clara Arrieta-Echeverri[1*], Geysson Javier Fernandez[2], Javier Correa-Álvarez[1]; Adriana Duarte-Riveros[3]; Jorge A. Bardales[3]; Diego Fernando Villanueva-Mejía[1]; Laura Sierra-Zapata[1]

*** Correspondence:**

Maria Clara Arrieta-Echeverri

marriet1@eafit.edu.co

Laura Sierra-Zapata

lsierra3@eafit.edu.co

Strain ID	Origin	Morphology	Geneious Taxa ID	GenBank Accession number
**T1.1**	Liquor	Yeast	*Pichia membranifaciens*	OR186528-OR186529
**T2.2**	Liquor	Yeast	*Pichia membranifaciens*	OR186604-OR186605
**T5.1**	Liquor	Bacteria	*Acetobacter*	OR228914-OR228915
**T8.2**	Liquor	Bacteria	*Lentilactobacillus hilgardii*	OR228929
**T9.2**	Liquor	Bacteria	*Lentilactobacillus buchneri*	OR228967-OR228968
**T12.2**	Liquor	Bacteria	*Lentilactobacillus hilgardii*	OR229410-OR229411
**T13.2**	Liquor	Bacteria	*Lentilactobacillus hilgardii*	OR229408-OR229409
**T14.1**	Liquor	Yeast	*Pichia kudriavzevii*	OR186604-OR186605
**T33.1**	Grains	Bacteria	*Lactobacillus hilgardii*	OR229145-OR229146
**T37**	Grains	Bacteria	*Acetobacter*	OR228906-OR228907
**T38.1**	Grains	Bacteria	*Lentilactobacillus hilgardii*	OR229141-OR229142
**T41.1**	Grains	Bacteria	*Lentilactobacillus hilgardii*	OR228986-OR228987
**T47**	Grains	Bacteria	*Acetobacter pasteurianus*	OR229145-OR229146
**T49.1**	Grains	Yeast	*Pichia membranifaciens*	OR186606-OR186607
**T49.2**	Grains	Bacteria	*Acetobacter*	OR228912-OR228913
**T59.1**	Grains	Bacteria	*Lentilactobacillus*	OR228920-OR228921
**T64.2**	Liquor	Bacteria	*Acetobacter sp.*	OR228924-OR228925
**T66.1**	Liquor	Bacteria	*Lentilactobacillus hilgardii*	OR229155-OR229156
**T78.3**	Liquor	Yeast	*Kazachstania exigua*	OR186608-OR186609
**T94.2**	Liquor	Bacteria	*Lentilactobacillus*	OR228969-OR228970
**T95.3**	Liquor	Bacteria	*Acetobacter tropicalis*	OR228984-OR228985
**T101.1**	Liquor	Bacteria	*Schleiferilactobacillus harbinensis*	OR204989-OR204990
**T101.3**	Liquor	Bacteria	*Acetobacter pomorum*	OR187893-OR187894

Table 1 (Additional File 1). Selection of identified isolates belonging to the Microbial collection of strains derived from the WK microbial consortium and their respective molecular identification with the NCBI accession numbers. To access files please follow the link **https://www.ncbi.nlm.nih.gov/nucleotide**/ and insert the respective **GenBank Accession number.**

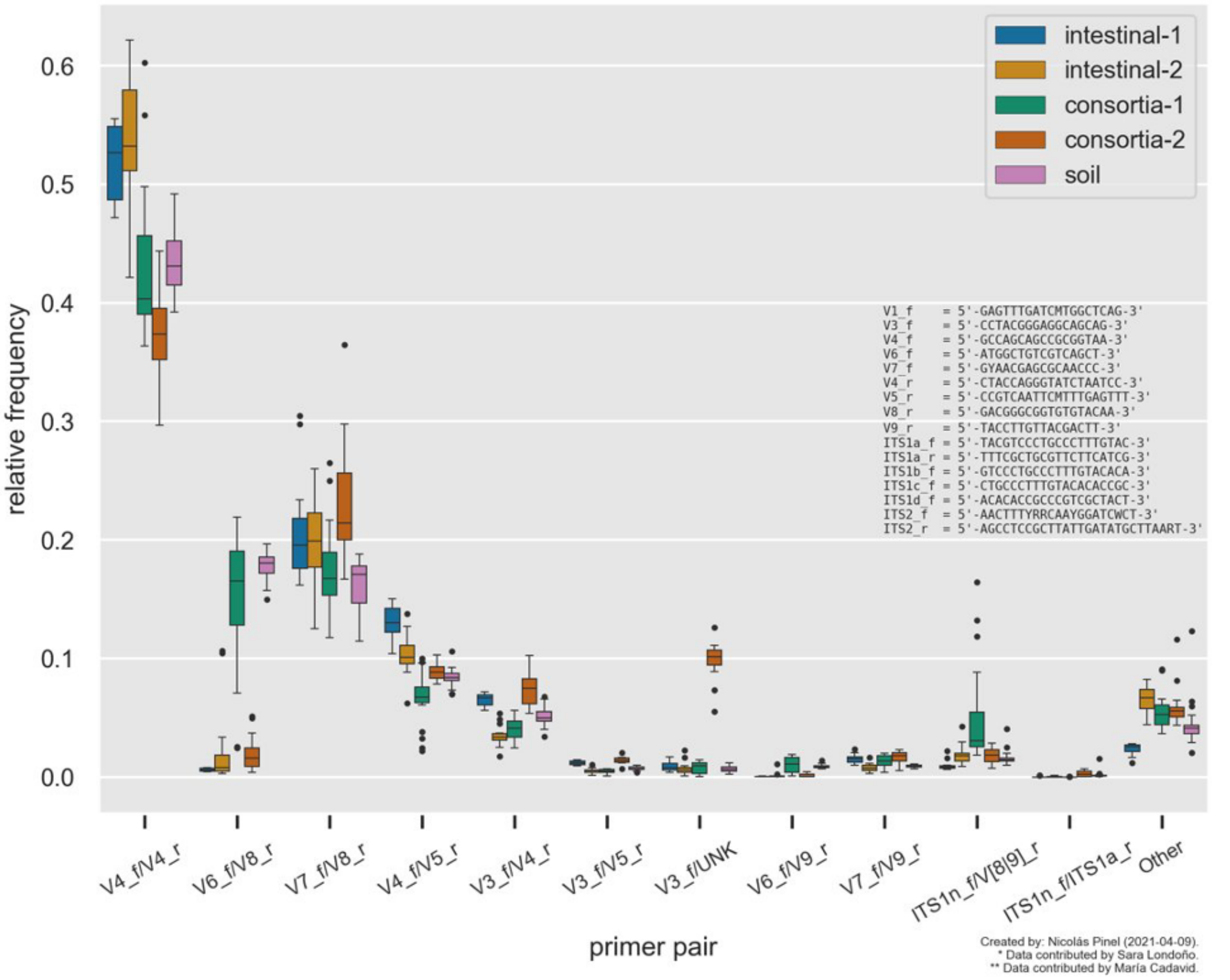

Figure 1 (Additional file 1). Primer-pair use frequency following our *in-house* source code of annotation. Primer frequencies correspond to those observed in 12 and 16* human stool (blue and ochre), 24 consortia-1, 13 consortia-2** (green and orange), and 35 soil samples (pink); each set was prepared and ran independently. Libraries were prepared with the Swift Amplicon 16S+ITS Panel kit and sequenced on iSeq 100 i1 Reagent v2 (300-cycle) cells.

Note: material was provided by researchers of the institution in which bioinformatic processes were carried out simultaneously but applied to different biological systems.

Sample	Library Code	Number of reads [bp]
Grain1	74isl	290932
Grain2	75isl	74878
FT 0h_1	76isl	125052
FT 0h_2	77isl	226426
FT 10h_1	78isl	151024
FT 10h_2	79isl	182280
FT 24h_1	80isl	285236
FT 24h_2	81isl	320976
FT 34h_1	82isl	115402
FT 34h_2	83isl	313936
FT 48h_1	84isl	235796
FT 48h_2	85isl	273638
FT 58h_1	86isl	226844
FT 58h_2	87isl	314748
FT 72h_1	88isl	185580
FT 72h_2	89isl	351840
FT 82h_1	90isl	326312
FT 82h_2	91isl	358246
FT 96h_1	92isl	321686
FT 96h_2	93isl	261754
FT 106h_1	94isl	313248
FT 106h_2	95isl	289282
FT 120h_1	96isl	356678
FT 120h_2	97isl	328494
	**Total**	6230288

Table 2 (Additional file 1). Metataxonomic sequencing data of the 24 samples from the water-kefir derived microbial consortium. Libraries were prepared using the SWIFT AMPLICON® 16S+ ITS PANEL protocol. Sequencing process was performed using the Iseq 100 system with a 2 x 150 bp read length at EAFIT University sequencing facility (AXOMICS)

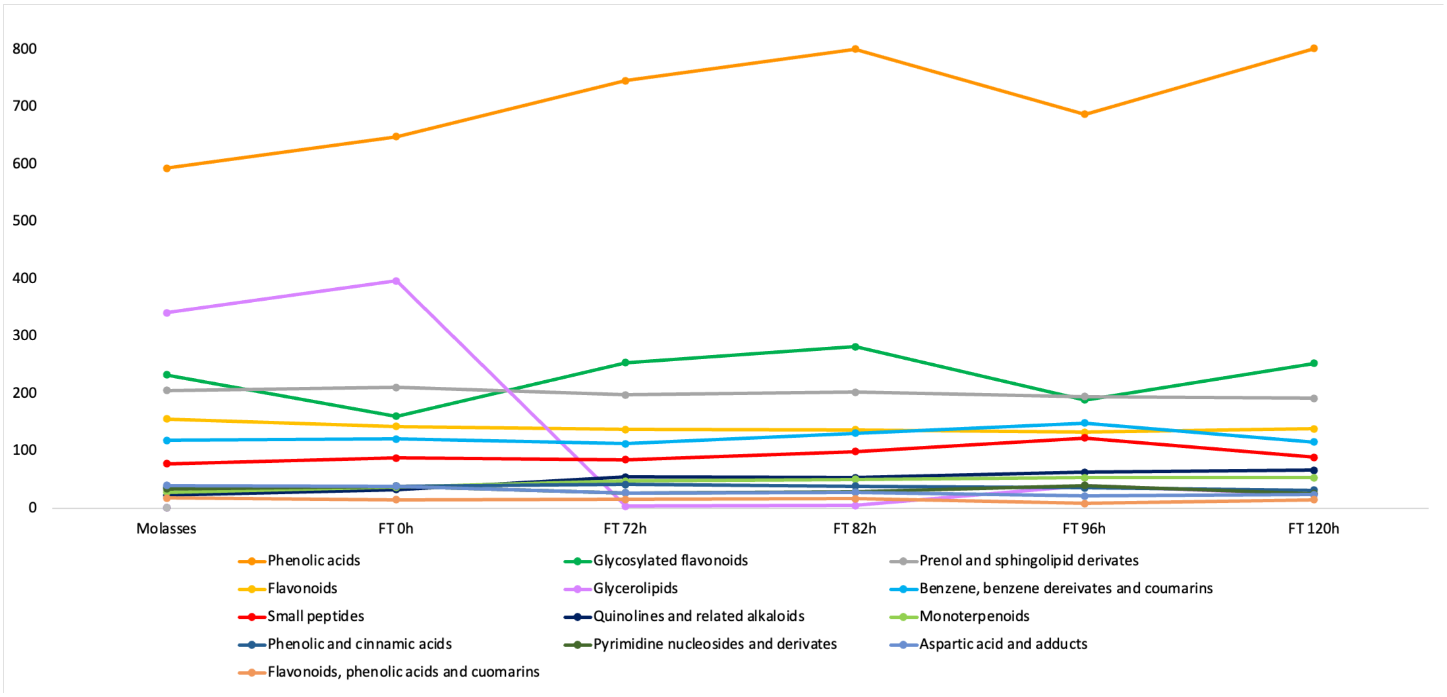

Figure 2 (Additional file 1). Relative abundance of the annotated chemical families along the fermentation course. Each line represents one identified chemical family.

Accession	Sample Name	SPUID	Organism	TaxID	BioProject
SAMN35984165	Grain1	Grain1	food metagenome	870726	PRJNA987609
SAMN35984166	Grain2	Grain2	food metagenome	870726	PRJNA987609
SAMN35984167	FT0h_1	FT0h_1	food metagenome	870726	PRJNA987609
SAMN35984168	FT0h_2	FT0h_2	food metagenome	870726	PRJNA987609
SAMN35984169	FT10h_1	FT10h_1	food metagenome	870726	PRJNA987609
SAMN35984170	FT10h_2	FT10h_2	food metagenome	870726	PRJNA987609
SAMN35984171	FT24h_1	FT24h_1	food metagenome	870726	PRJNA987609
SAMN35984172	FT24h_2	FT24h_2	food metagenome	870726	PRJNA987609
SAMN35984173	FT34h_1	FT34h_1	food metagenome	870726	PRJNA987609
SAMN35984174	FT34h_2	FT34h_2	food metagenome	870726	PRJNA987609
SAMN35984175	FT48h_1	FT48h_1	food metagenome	870726	PRJNA987609
SAMN35984176	FT48h_2	FT48h_2	food metagenome	870726	PRJNA987609
SAMN35984177	FT58h_1	FT58h_1	food metagenome	870726	PRJNA987609
SAMN35984178	FT58h_2	FT58h_2	food metagenome	870726	PRJNA987609
SAMN35984179	FT72h_1	FT72h_1	food metagenome	870726	PRJNA987609
SAMN35984180	FT72h_2	FT72h_2	food metagenome	870726	PRJNA987609
SAMN35984181	FT82h_1	FT82h_1	food metagenome	870726	PRJNA987609
SAMN35984182	FT82h_2	FT82h_2	food metagenome	870726	PRJNA987609
SAMN35984183	FT96h_1	FT96h_1	food metagenome	870726	PRJNA987609

SAMN35984184	FT96h_2	FT96h_2	food metagenome	870726	PRJNA987609
SAMN35984185	FT106h_1	FT106h_1	food metagenome	870726	PRJNA987609
SAMN35984186	FT106h_2	FT106h_2	food metagenome	870726	PRJNA987609
SAMN35984187	FT120h_1	FT120h_1	food metagenome	870726	PRJNA987609
SAMN35984188	FT120h_2	FT120h_2	food metagenome	870726	PRJNA987609

Table 3 (Additional file 1). Accession number list for the 24 available sequence data obtained for the compositional metagenomics analysis (metataxonomics) of WK. Data available at the NCBI repository (https://www.ncbi.nlm.nih.gov/biosample/).
